# A Triple Combination of Targeting Ligands Increases the Penetration of Nanoparticles across a Blood-Brain Barrier Culture Model

**DOI:** 10.3390/pharmaceutics14010086

**Published:** 2021-12-30

**Authors:** Szilvia Veszelka, Mária Mészáros, Gergő Porkoláb, Anikó Szecskó, Nóra Kondor, Györgyi Ferenc, Tamás F. Polgár, Gábor Katona, Zoltán Kóta, Lóránd Kelemen, Tibor Páli, Judit P. Vigh, Fruzsina R. Walter, Silvia Bolognin, Jens C. Schwamborn, Jeng-Shiung Jan, Mária A. Deli

**Affiliations:** 1 Biological Research Centre, Institute of Biophysics, Eötvös Loránd Research Network, Temesvári krt. 62, H-6726 Szeged, Hungary; meszaros.maria@brc.hu (M.M.); porkolab.gergo@brc.hu (G.P.); szecskoaniko@gmail.com (A.S.); knorka98@gmail.com (N.K.); polgar.tamas@brc.hu (T.F.P.); kota.zoltan@brc.hu (Z.K.); kelemen.lorand@brc.hu (L.K.); pali.tibor@brc.hu (T.P.); vigh.judit@brc.hu (J.P.V.); walter.fruzsina@brc.hu (F.R.W.); 2 Doctoral School of Biology, University of Szeged, Dugonics tér 13, H-6720 Szeged, Hungary; 3 Biological Research Centre, Institute of Plant Biology, Eötvös Loránd Research Network, Temesvári krt. 62, H-6726 Szeged, Hungary; ferenc.gyorgyi@brc.hu; 4 Faculty of Pharmacy, Institute of Pharmaceutical Technology and Regulatory Affairs, University of Szeged, Eötvös u. 6, H-6720 Szeged, Hungary; katona.gabor@szte.hu; 5 Luxembourg Centre for Systems Biomedicine (LCSB), Developmental and Cellular Biology, University of Luxembourg, 4365 Belvaux, Luxembourg; silvia.bolognin@uni.lu (S.B.); jens.schwamborn@uni.lu (J.C.S.); 6 Department of Chemical Engineering, National Cheng Kung University, Tainan 70101, Taiwan; jsjan@ncku.edu.tw

**Keywords:** blood-brain barrier, brain endothelial cell, drug targeting, nanoparticle, triple targeting ligand, solute carrier, glutathione, leucine, ascorbic acid, brain organoid

## Abstract

Nanosized drug delivery systems targeting transporters of the blood-brain barrier (BBB) are promising carriers to enhance the penetration of therapeutics into the brain. The expression of solute carriers (SLC) is high and shows a specific pattern at the BBB. Here we show that targeting ligands ascorbic acid, leucine and glutathione on nanoparticles elevated the uptake of albumin cargo in cultured primary rat brain endothelial cells. Moreover, we demonstrated the ability of the triple-targeted nanovesicles to deliver their cargo into midbrain organoids after crossing the BBB model. The cellular uptake was temperature- and energy-dependent based on metabolic inhibition. The process was decreased by filipin and cytochalasin D, indicating that the cellular uptake of nanoparticles was partially mediated by endocytosis. The uptake of the cargo encapsulated in triple-targeted nanoparticles increased after modification of the negative zeta potential of endothelial cells by treatment with a cationic lipid or after cleaving the glycocalyx with an enzyme. We revealed that targeted nanoparticles elevated plasma membrane fluidity, indicating the fusion of nanovesicles with endothelial cell membranes. Our data indicate that labeling nanoparticles with three different ligands of multiple transporters of brain endothelial cells can promote the transfer and delivery of molecules across the BBB.

## 1. Introduction

The penetration of most therapeutics, especially large biomolecules, into the brain is actively blocked by the blood-brain barrier (BBB), making pharmaceutical treatment of central nervous system (CNS) disorders difficult [1]. Nanoparticles (NPs) are promising tools to solve these unmet therapeutic needs. Drugs encapsulated in nanovesicles, like liposomes, are already used clinically to treat systemic diseases, especially tumors [2]. However, in the case of the CNS, there are not yet NP-formulated drugs available [3]. NPs enhance the crossing of drug molecules through biological barriers while decreasing unwanted side effects via entrapment of the active agent. It needs to be highlighted that encapsulation of drugs by nanoparticles alone is not sufficient for CNS delivery; decoration of the surface of NPs with targeting ligands is necessary. Targeting BBB transporters is a successful strategy to increase brain penetration of biomolecules [4]. A large number of transporters are expressed on the plasma membrane of brain endothelial cells, which exhibit an expression pattern specific for the BBB [5,6,7], and are potential candidates as BBB-targeting ligands for NPs. Tripeptide glutathione (GSH) is one of the most successful BBB-targeting ligands, although its transporter has not been identified. Liposomes targeted with GSH and loaded with doxorubicin cargo were effective in inhibiting tumor growth in a mouse glioma model [8], and these NPs were also investigated in phase I/II clinical trials [9].

The biggest superfamily of transporters on brain endothelial cells is that of solute carriers (SLC), which provide nutrients to brain cells [10]. SLCs actively transport well-known therapeutic drugs across the BBB; for example, in the treatment of Parkinson’s disease, the LAT1/SLC7A5 transporter carries L-DOPA to the brain [4]. The number of clinical trials with drugs and biopharmaceutics developed for targeting SLCs is increasing, but SLC transporters as molecular targets in general and at the BBB are under-researched [11]. We revealed the specific expression pattern of SLC genes in primary rat brain endothelial cells and demonstrated that among the BBB influx transporters, the expression levels of glucose transporter 1 (GLUT1/SLC2A1) and large amino acid transporter 1 (LAT1/SLC7A5) were among the highest [7], so these can potentially be exploited to target NPs. In our previous study, we systematically tested vesicular NPs made from non-ionic surfactants (niosomes), labeled with a single or dual combination of three different ligands targeting nutrient transporters of the BBB [12]. We demonstrated that the combination of alanine and glutathione significantly increased the uptake and permeability of the NPs across the in vitro model of the BBB compared to the non-targeted or single-targeted NPs [12,13]. Dual-targeting of NPs also elevated the brain penetration of the encapsulated cargo in mice [12]. Furthermore, the delivery of a large protein cargo was enhanced by alanine-glutathione dual-targeted NPs not only into cultured brain endothelial cells but also into pericytes, astrocytes and neurons [13]. In our most recent work, we investigated the initial docking step between the targeting ligand glutathione and the cell membrane of living brain endothelial cells. Using an innovative and novel optical tweezer-based method, we measured the adhesion forces between glutathione and cells and demonstrated that it is in the low pN range [14]. 

Based on our previous results, we hypothesize that an optimal combination of different SLC ligands would lead to better docking of NPs to the surface of brain endothelial cells resulting in the shuttle of NPs and their cargo across the BBB and increased brain delivery. In our present study, we designed niosomes with a new triple combination of BBB transporter ligands. We selected leucine, the ligand of LAT1, vitamin C, the ligand of sodium-ascorbate cotransporter (SVCT2/SLC23A2) and glutathione to label the surface of NPs. As model biomolecule cargo, we used bovine serum albumin labeled with Texas Red. The NPs were characterized and tested in uptake, permeability and uptake mechanism experiments using our well-characterized co-culture BBB model. Furthermore, we tested whether the NPs that cross the BBB model enter into midbrain-like organoids generated from healthy and Parkinson’s disease patient-specific stem cells.

## 2. Materials and Methods

### 2.1. Animals

For primary cell isolations, brain tissues were obtained from 3-week-old and newborn outbred Wistar rats (Harlan Laboratories, Envigo, Indianapolis, IN, USA) of both sexes. The animals were fed on standard rodent chow and water ad libitum and were kept under a 12 h light/dark cycle in the conventional animal house of the Biological Research Centre, Szeged. Organ harvest from animals was performed following the regulations of the 1998 XXVIII Hungarian law and the EU Directive 2010/63/EU about animal protection and welfare.

### 2.2. Materials and Reagents

All reagents were purchased from Merck Life Science Kft., Budapest, Hungary, unless otherwise indicated.

### 2.3. Cell Cultures

Isolation of primary rat brain endothelial cells (RBECs), pericytes and astroglia were performed according to the method described in our previous studies [15,16]. After isolation, RBECs were seeded onto culture dishes (Corning Costar, New York, NY, USA) coated with collagen type IV (100 μg/mL) and fibronectin (25 μg/mL) and were cultured in a BBB cell culture medium containing DMEM/HAM’s F-12 (Gibco, Waltham, MA, USA), supplemented with 15% plasma-derived bovine serum (PDS, First Link, Wolverhampton, UK), 10 mM HEPES, 100 μg/mL heparin, 5 μg/mL insulin, 5 μg/mL transferrin, 5 ng/mL sodium selenite (ITS, Pan-Biotech, Aidenbach, Germany), 1 ng/mL basic fibroblast growth factor (bFGF) and 50 μg/mL gentamicin at 37 °C in a humidified incubator with 5% CO_2_. During the first 3 days of culture, the medium of endothelial cells also contained 3 μg/mL puromycin to eliminate P-glycoprotein negative, contaminating cell types [17]. After the first 3 days of culture, the amount of PDS was decreased from 15% to 10% in the culture medium. After isolation, pericytes were seeded onto culture dishes (VWR International, Radnor, PA, USA) coated with collagen type IV (100 μg/mL), whereas astrocytes were plated onto uncoated 75 cm^2^ flasks (TPP, Trasadingen, Switzerland). Both pericytes and astrocytes were cultured in DMEM medium (low glucose, Gibco, subsidiary of Merck Life Sciences) supplemented with 10% fetal bovine serum (FBS, Pan-Biotech, Aidenbach, Germany) and 50 μg/mL gentamicin. 

Midbrain organoids were generated from human floor plate neuronal progenitor cells as described previously in detail [18]. Organoids were established using healthy (ID number: #232) and Parkinson’s disease patient (ID number: #317) specific stem cells harboring a triplication in the SNCA gene [19]. First, 9 × 10^3^/well progenitor cells were seeded into a 96-well low-attachment plate (Corning Costar). Cells were first kept in maintenance medium for a week followed by a two-step patterning for a total of 9 days, as described earlier [18]. Next, the patterning organoids were washed in Dulbecco’s Modified Eagle Medium/Nutrient Mixture F-12 (DMEM/F12) and were gently seeded into maturation medium based on a DMEM/F12 and Neurobasal medium (Thermo Fisher Scientific, Waltham, MA, USA) 1:1 ratio mixture. This was supplemented with gentamicin (50 µg/mL), L-glutamine (2 mM, Thermo Fisher Scientific), B27 supplement (100×, Thermo Fisher Scientific), N2 supplement (200×, Thermo Fisher Scientific), dibutyryl-cAMP (500 µM), brain-derived neurotrophic factor (BDNF, 10 ng/mL, Peprotech, Cranbury, NJ, USA), glial cell-derived neurotrophic factor (GDNF, 10 ng/mL, Peprotech), transforming growth factor-β3 (TGF-β3, 1 ng/mL, Peprotech), Activin A (2.5 ng/mL, Peprotech), L-ascorbic acid (200 µM) and dual antiplatelet therapy (DAPT, 10 µM, Tocris Bioscience, Bristol, UK). The maturation medium on the organoids was changed three times per week. Before the assembly of the BBB-organoid co-culture model, organoids were first embedded into growth factor-reduced Matrigel (Corning) at the bottom of a 12-well plate (Corning Costar) as follows: the 120-day-old organoids were washed in DMEM/F12, then were placed into a 10 µL volume Matrigel droplet. These were left to solidify for 12 min at 37 °C; then wells were filled up with the maturation medium. The next day the organoid-BBB model was assembled by adding a 1:1 mixture of BBB cell culture and maturation medium to the bottom compartment and kept under this condition until use. Top compartments above the brain endothelial cells received BBB cell culture medium.

### 2.4. Synthesis of the Targeting Ligands of Niosomes

Among the targeting ligands, the dodecanoyl leucine and DSPE-PEG-glutathione were synthesized in our institute and the 6-O-palmitoyl-L-ascorbic acid was purchased from Merck Life Science. For the synthesis of DSPE-PEG-glutathione, 13.5 mg glutathione (0.044 mM) was reacted with 100 mg DSPE-PEG-maleimide (N-[(3-maleimide-1-oxopropyl) aminopropyl polyethyleneglycol-carbamyl] distearoylphosphatidyl-ethanol-amine; DSPE-PEG-MAL; 0.035 mM) in 0.1 M ammonium acetate for a day under nitrogen. The product was lyophilized three times to remove ammonium acetate [12,13]. Dodecanoyl leucine was prepared in an analogous way to the synthesis of dodecanoyl alanine [12]. Briefly, 10 mL NaOH (1 M) and 0.197 g (1.5 mmol) L-leucine were added into a one-neck flask. After the system was cooled to 0 °C, 0.33 mL (1.4 mmol) dodecanoyl chloride was added dropwise to the mixture and maintained for 5 h at 0 °C. Then, 1.6 mL hydrochloric acid (12 M) was added to the reaction, and the white precipitate was filtrated. Finally, the product was washed three times with deionized water and dried at 45 °C for 24 h.

### 2.5. Preparation of Niosomes

Niosomes were prepared as described in our previous paper with minor modifications [12]. For the preparation of non-targeted niosomes (N), non-ionic surfactants Span 60 (sorbitan-monostearate) and Solulan C24 (cholesteryl-poly-24-oxyethylene-ether, Chemron Co., Paso Robles, CA, USA), as well as cholesterol, were dissolved in a hot mixture of chloroform and ethanol (1:2) in a round-bottom flask. To prepare ascorbic acid-glutathione-leucine targeted niosomes (N-AGL), 6-O-palmitoyl-L-ascorbic acid (4 *w*/*w*% of total lipid), DSPE-PEG-GSH (4 *w*/*w*% of total lipid) and dodecanoyl-leucine (4 *w*/*w*% of total lipid) were also added to the mixture before the dissolving step. The removal of organic solvents by a vacuum pump yielded a thin lipid film layer. The dry lipid film was hydrated with 10 mL phosphate buffer (PBS; KCl 2.7 mM, KH_2_PO_4_ 1.5 mM, NaCl 136 mM, Na_2_HPO_4_ × 2 H_2_O 6.5 mM, pH 7.4) containing 0.1 mg/mL Texas red-labeled bovine serum albumin (TR-BSA, 67.12 kDa, Thermo Fischer Scientific, Waltham, MA, USA). After the hydration step, the mixtures were sonicated in a water bath for 60 min at 45 °C. To obtain vesicles, the hot suspension was forced through syringe filters with 0.45 µm, then 0.22 µm pore size (Filtropur, Sarstedt, Germany) in sequential steps. The non-encapsulated cargo was removed by ultracentrifugation (123,249× *g*, 4 °C, 3 h), the pelleted niosomes were resuspended in PBS for size and zeta potential measurements or in phenol red-free DMEM/HAM’s F-12 medium for experiments and stored at 4 °C.

### 2.6. Characterization of Niosomes

Nanovesicle samples were evaluated under a transmission electron microscope (TEM, JEM-1400 Flash, JEOL Ltd., Tokyo, Japan) to identify the morphological characteristics of the particles. Samples (20 μL, 50 mg/mL) were dropped on formvar-coated 150-mesh copper grids, and excessive fluid was removed with the edge of a filter paper after 1 min. Samples were contrasted with 10 μL uranyl acetate (0.5%, Electron Microscopy Sciences) in 50% ethanol for 3 min (2 times) and were dried under a Petri dish overnight before the electron microscopic evaluation. The negatively stained nanoparticles were localized with JEM-1400 Flash TEM (JEOL) at 10,000× magnification. Tag Image File Format (TIFF) images were recorded at 10,000 and 60,000× magnification with the built-in Matataki Flash camera of the TEM.

Particle size, polydispersity index (PDI) and zeta potential of niosomes were measured by dynamic light scattering (Malvern Zetasizer Nano ZS, equipped with a He-Ne laser (λ = 632.8 nm), Malvern Instruments, UK). Before measurements, samples were diluted in PBS to a final concentration of 2 mg/mL. Means were calculated from the average of at least 3 × 13 measurements per sample. To determine the amount of encapsulated model biomolecule in the NPs after ultracentrifugation, the TR-BSA was measured from the supernatant and the pellet of niosomes releasing the cargo by 50% *v*/*v* ethanol and measured by spectrofluorometer (Fluorolog 3, Horiba Jobin Yvon, Palaiseau, France) at 592 nm excitation and 612 nm emission wavelengths. The fluorescence values were extrapolated to concentrations from a standard calibration curve. The encapsulation efficiency % (EE%) and loading capacity (LC) were calculated by the following equations:

$$\mathrm{EE}\%=\frac{\mathrm{Amount}\;\mathrm{of}\;\mathrm{TR}-\mathrm{BSA}\;\mathrm{in}\;\mathrm{the}\;\mathrm{NP}\;\mathrm{sample}}{\mathrm{Amount}\;\mathrm{of}\;\mathrm{total}\;\mathrm{TR}-\mathrm{BSA}\;}\times 100$$

$$\mathrm{LC}\%=\frac{\mathrm{Amount}\;\mathrm{of}\;\mathrm{encapsulated}\;\mathrm{TR}-\mathrm{BSA}\;\mathrm{in}\;\mathrm{the}\;\mathrm{NP}\;\mathrm{sample}}{\mathrm{The}\;\mathrm{weight}\;\mathrm{of}\;\mathrm{total}\;\mathrm{lipids}\;\mathrm{in}\;\mathrm{the}\;\mathrm{NPs}}\times 100$$

In order to investigate the in vitro cargo release of TR-BSA-loaded NPs, the modified paddle method (Hanson SR8 Plus, Teledyne Hanson Research, Chatsworth, CA, USA) was applied. The experiment was carried out at 37 °C under 50 rpm constant stirring. From the investigated formulations and reference solution, 1-1 mL were placed in immersion cells (Agilent, Santa Clara, CA, USA) before experiment, and 25 mL PBS (pH 7.4) was used as a release medium. The Teflon immersion cell of 1.77 cm^2^ surface area consisted of a cap, an artificial polystyrene membrane (Isopore, VMTP, Millipore, subsidiary of Merck Life Sciences) with 0.05 µm pore size fixed with an O-ring. Aliquots (1 mL) were withdrawn from the release medium at predetermined time intervals up to 4 h and then replaced with an equivalent volume of the fresh release medium to maintain sink condition. Quantification of aliquots was performed by spectrofluorometer (Fluorolog 3, Horiba Jobin Yvon, France) at 592 nm excitation and 612 nm emission wavelengths. Three parallel measurements were performed with both reference and formulation data presented as mean ± SD.

### 2.7. Cell Viability Assay

Kinetics of primary RBEC responses to niosomes were monitored by impedance measurement (RTCA-SP; Agilent Technologies, Santa Clara, CA, USA), which is label-free, real-time, non-invasive and correlates linearly with the viability of cells [20,21]. After background measurements with culture medium, RBECs were seeded at a density of 6 × 10^3^ cells/well in 96-well plates with integrated gold electrodes (E-plate 96, Agilent), coated with collagen type IV (100 µg/mL) and fibronectin (25 µg/mL). When the impedance of cells reached a plateau phase indicating a confluent culture, cells were treated with niosomes (0.3, 1 or 3 mg/mL), and their impedance was monitored every 5 min for 24 h. Cell index was defined as R_n_-R_b_ at each time point of measurement, where R_n_ is the impedance of the well when it contains adherent cells, and R_b_ is the background impedance of the well containing culture medium alone. Cell index was normalized in each well to the value measured at the last timepoint before the treatment.

### 2.8. Cellular Uptake Studies

For investigation of cellular uptake, RBECs were cultured in 24-well plates (3 × 10^4^ cells/well, Corning Life Scinces, Tewksbury, MA, USA) coated with collagen type IV (100 μg/mL) and fibronectin (25 μg/mL). Confluent monolayers were incubated with TR-BSA (3 µg/mL) or niosomes (N or N-AGL, 1 mg/mL containing 3 μg/mL TR-BSA cargo) diluted in RBEC culture medium at 37 °C for 8 h or 24 h in a CO_2_ incubator. To elucidate the uptake mechanisms, cells were incubated with N-AGL at 4 °C for 4 h, co-incubated with niosomes and metabolic inhibitor sodium azide (1 mg/mL) at 37 °C for 4 h, or pre-treated with endocytosis inhibitors filipin (5 μg/mL, 15 min) or cytochalasin D (0.125 μg/mL, 1 h) and then incubated with niosomes at 37 °C for 4 h. To study the role of the surface charge in the cellular uptake of niosomes, we treated the cells with cationic lipid TMA-DPH (1-(4-trimethylammoniumphenyl)-6-phenyl-1,3,5-hexatriene, 54 μM; Molecular Probes, Life Technologies) for 30 min or digested the surface glycocalyx of RBECs with neuraminidase (1 U/mL, 1-h pretreatment) before the uptake of N-AGL (1 mg/mL, 37 °C, 4 h). After incubation with niosomes, cells were washed three times with ice-cold PBS supplemented with 0.1% BSA, once with acid stripping buffer (glycine 50 mM, NaCl 100 mM, pH 3) to remove cell surface-associated niosomes and once with PBS. Finally, cells were lysed in distilled water containing 10 mg/mL Triton X-100 detergent, and the fluorescent signal of the TR-BSA cargo was quantified with a spectrofluorometer (Fluorolog 3, Horiba Jobin Yvon) at 592 nm excitation and 612 nm emission wavelengths. The cellular amount of cargo after uptake experiments was normalized to the protein content of wells measured by BCA Protein Assay (Thermo Fischer Scientific, USA).

For visualization of the cellular uptake, RBECs were cultured on glass coverslips (10^5^ cells/coverslip, borosilicate; VWR, USA) placed in 24-well culture dishes. Confluent monolayers were incubated with either TR-BSA solution (3 μg/mL) or niosomes (1 mg/mL N or N-AGL containing 3 μg/mL cargo) diluted in RBEC culture medium at 37 °C for 24 h in a CO_2_ incubator. After the incubation, culture medium was completely removed, cells were washed three times with Ringer-HEPES buffer (118 mM NaCl, 4.8 mM KCl, 2.5 mM CaCl_2_, 1.2 mM MgSO_4_, 5.5 mM D- glucose, 20 mM HEPES, pH 7.4) supplemented with 1% PDS and fixed with 1% paraformaldehyde solution (10 min at room temperature). For the staining of cell nuclei, Hoechst 33342 dye (1 μg/mL, 10 min) was used. After mounting the samples (Fluoromount-G, Southern Biotech, Birmingham, AL, USA), TR-BSA cargo inside cells was imaged using the 543 nm laser line on a Leica TCS SP5 confocal laser scanning microscope (Leica Microsystems, Wetzlar, Germany) equipped with an HC PL APO 20× objective (NA = 0.7). Z-stacks of 10 images with an average thickness of 0.4–0.5 μm per slice were generated from 8–10 non-overlapping visual fields by the maximum projection of images. Mean fluorescence intensity values of images were determined using the mean gray value function on the 543 nm channel in Fiji, a distribution of ImageJ2 [22].

### 2.9. Measurement of Plasma Membrane Fluidity

RBECs grown in culture dishes were treated with N or N-AGL (1 mg/mL) diluted in culture medium for 4 h at 37 °C in a CO_2_ incubator. The cells were washed twice with PBS, collected by trypsinization, and resuspended in Ringer-Hepes buffer. The density of cells was set by absorbance measurement to OD360 = 0.1 (Helios Epsilon Spectrophotometer, Thermo Fischer Scientific, USA). Cells were labeled with 0.2 μM TMA-DPH for 5 min. Fluorescence anisotropy was measured with a spectrofluorometer supplemented with a T-format monochromator (Fluorolog 3, Horiba Jobin Yvon). Excitation and emission wavelengths were 360 and 430 nm, respectively (5 nm and 5 nm slits). Cells were kept at 37 °C under stirring conditions. Anisotropy data were acquired every second for 5 min, then benzyl alcohol (50 mM), a strong membrane fluidizer, was added, and data were acquired for another 5 min. The average of 50 anisotropy measurements in the last 1 min of each treatment was calculated and compared [23,24].

### 2.10. Permeability Studies

For permeability studies, we used a well-characterized triple co-culture BBB model in which RBECs, brain pericytes and astrocytes are cultured together in a Transwell system [7,15]. Astroglial cells were passaged (5 × 10^4^ cells/well) to 24-well plates (Corning Costar, USA) coated with collagen type IV (100 μg/mL). To prepare the co-culture model, pericytes at P2 were passaged (5 × 10^3^ cells/insert) to the bottom side of tissue culture inserts (Transwell, polycarbonate membrane, 3 μm pore size, surface 0.33 cm^2^; Corning Costar, USA) coated with collagen type IV (100 μg/mL). RBECs were seeded (3 × 10^4^ cells/insert) to the upper side of the culture insert membrane coated with Matrigel (growth factor reduced). Then, the inserts containing RBECs and brain pericytes on the two sides of the membrane were placed on 24-well plates containing astrocytes at the bottom. The three cell types were cultured together for 4 days before permeability experiments began. Both the upper and lower fluid compartments of the model received endothelial cell culture medium supplemented with 550 nM hydrocortisone. For the last 24 h before and during the experiment, the upper compartment of the model was also supplemented with 250 μM 8-(4-chlorophenylthio)adenosine 3′,5′-cyclic monophosphate (cPT-cAMP) and with 17.5 μM Ro-20-1724, a selective inhibitor of cAMP-specific phosphodiesterase to increase the tightness of the barrier [17,25].

The tightness of the BBB model was verified by measurements of transendothelial electrical resistance (TEER) by an EVOM voltohmmeter (World Precision Instruments, Sarasota, FL, USA) combined with STX-2 electrodes. When TEER values reached a plateau level (203 ± 46 Ω × cm^2^; *n* = 24), the model was used for experiments. The upper donor compartment (0.2 mL) was incubated with either TR-BSA solution (3 μg/mL) or niosomes (N or N-AGL, 1 mg/mL containing 3 μg/mL TR-BSA cargo) diluted in phenol red-free DMEM/HAM’s F-12 medium (Gibco) supplemented with 1% PDS at 37 °C for 2, 4, 8 and 24 h on a horizontal shaker (150 rpm) in a CO_2_ incubator. The paracellular permeability marker molecule, 70 kDa FITC-dextran (10 μg/mL), was co-administered with free cargo or nanoparticles to verify the BBB integrity. After incubation, samples were collected from both compartments, and the fluorescent signal of TR-BSA cargo was quantified at 592 nm excitation and 612 nm emission wavelengths with the spectrofluorometer.

The apparent permeability coefficients (P_app_) were calculated from the cumulative clearance values of TR-BSA cargo sampled at multiple time points (2, 4, 8 and 24 h), as described previously [25,26]. Briefly, clearance values were calculated for each time point using the following equation:

$$\mathrm{clearance}\;\left(\mu L\right)=\frac{{\left[C\right]}_{A}{\;\times \;V}_{A}}{\;{\left[C\right]}_{D}}$$

where [C]_A_ and [C]_D_ are the concentrations of cargo in the acceptor and donor compartments, respectively, and V_A_ is the volume of the acceptor compartment (0.9 mL). Then, cumulative clearance values were plotted against time (min), and the clearance rate (μL/min) was determined as the slope of the line using linear regression. Finally, P_app_ values (cm/s) were calculated using the following equation:

$$P_{\mathrm{app}}\;\left(\mathrm{cm}/s\right)=\frac{\mathrm{Slope}}{A\times t}$$

by dividing the clearance rate (μL/min) with the culture insert surface area (0.33 cm^2^) and time (in seconds).

In a second experimental setup, cell culture inserts with brain endothelial cells and pericytes co-cultured with astroglia cells for 4 days were placed into 12-well plates containing midbrain-specific organoids derived from Parkinson’s disease patients (PD) and healthy control (Control) in the bottom. Donor compartments were treated with N or N-AGL (1 mg/mL for 24 h), similarly as we have already described. After incubation, the organoids were fixed with 1% of paraformaldehyde in PBS (4 °C, overnight), then the samples were cryoprotected with 30% sucrose solution (4 °C, overnight). Organoids were embedded into OCT (Tissue-Tek, Sakura Finetek, Torrance, CA, USA), then 20 µm thin sections were prepared with cryostat (Leica CM 1860, Leica Microsystems, Germany). Sections were permeabilized with 0.2% Triton X-100 in PBS for 10 min, then 2% normal horse serum and 0.3% BSA in PBS were used to block non-specific binding sites (4 °C, 60 min). Afterward, samples were incubated with mouse anti-βIII-tubulin antibody (Thermo Fisher Scientific) diluted to 1:1000 in blocking solution at 4 °C overnight. Then, samples were incubated with donkey anti-mouse Alexa 488 secondary antibody (Thermo Fisher Scientific) diluted to 1:400 and Hoechst 33342 for nuclei staining (1 µg/mL) in PBS (1 h, room temperature). Samples were washed with PBS after each step and mounted in a mounting medium. Staining of niosomes and βIII-tubulin were visualized with Leica TCS SP5 confocal laser scanning microscope. Z-stacks of 10 images with an average thickness of 0.6–0.7 μm per slice were generated from 8–10 non-overlapping visual fields by the maximum projection of images. To account for tissue autofluorescence, background subtraction was applied for all images of the 543 nm channel. To account for image-to-image variability in the organoid area, the mean fluorescence intensity values of the 543 nm channel images (determined using the Mean gray value function in Fiji) were divided by the area of organoids in the 488 nm channel (determined using the area function after thresholding in Fiji). 

### 2.11. Statistical Analysis

Data are presented as means ± SD. Statistical analyses were performed using GraphPad Prism 8 software (GraphPad Software, San Diego, CA, USA). Means were compared using Student’s *t*-test or one-way ANOVA followed by Dunnett’s posttest. Differences were considered statistically significant at *p* < 0.05. All experiments were repeated at least two times, and the number of parallel samples in each experiment was 4–8.

## 3. Results

### 3.1. Characterization of the Nanoparticles

**Figure 1:**
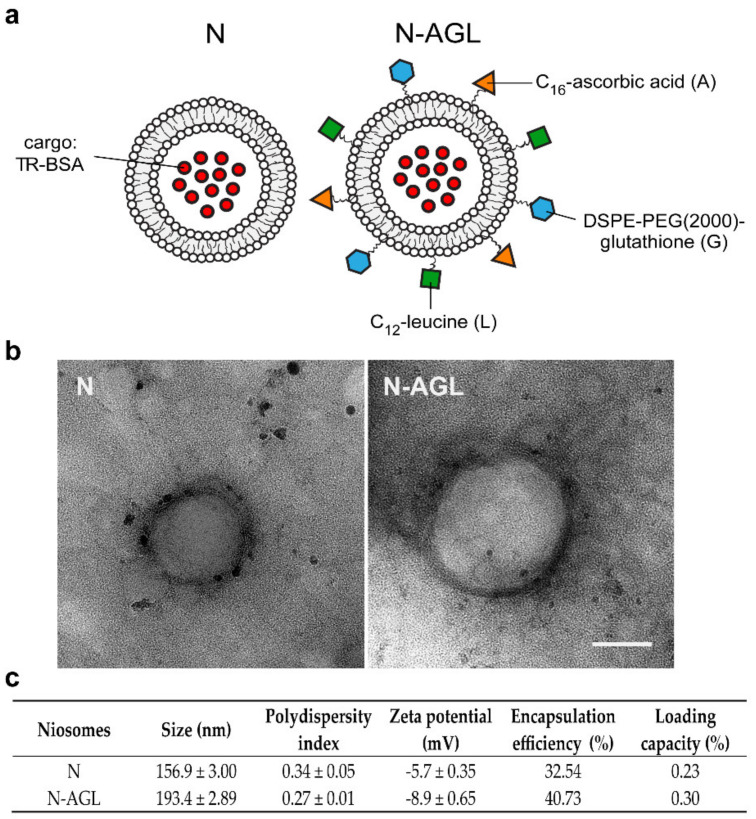
(**a**) Schematic drawing of non-targeted (N) and ascorbic acid-glutathione-leucine targeted (N-AGL) nanoparticles. TR-BSA: Texas red-labeled bovine serum albumin. (**b**) TEM images of nanoparticles. Bar: 100 nm. (**c**) Main physico-chemical properties of the nanoparticles. Values presented are means ± SD.

A schematic drawing of N and triple-targeted N-AGL nanovesicles are presented in Figure 1a. The morphology and stability of niosomes were well characterized in our previous articles [12,13]. The morphology of the nanovesicles was spherical as observed by TEM (Figure 1b). These results were in agreement with our previous study [12], where both TEM and AFM images of our nanoparticles were demonstrated. The mean diameters of non-targeted NPs were 157 nm and 193 nm in the case of the N-AGL group. The polydispersity index values were low (≤0.34) in both groups indicating a relatively narrow size distribution of the particles (Figure 1c). The surface charge of both the non-targeted NPs and targeted particles was slightly negative, −5.7 mV and 8.9 mV, respectively (Figure 1c), in agreement with our previous results [12,13]. The encapsulation efficiency of the large cargo TR-BSA was in the range of 32–40% (Figure 1c). In the case of non-targeted NPs, the loading capacity was 0.23%, and for the N-AGL group, it was 0.3% (Figure 1c). The in vitro releases of TR-BSA from the targeted and non-targeted formulations were compared with the TR-BSA solution. TR-BSA demonstrated the highest dissolution rate in PBS (62%) after 4-h incubation. The dissolution rates of TR-BSA from non-targeted and targeted NPs were 53% and 40%, respectively, indicating that targeted nanoparticles had a slower release profile.

Regarding the ligand density, based on the radius of triple-targeted NPs (97 nm), we calculated the surface area of the particles as 120 × 10^3^ nm^2^. The size of lipid head groups in niosomes is in the range of 0.25–0.5 nm^2^ [27]. Using these data, the number of lipid molecules in N-AGLs was estimated as 240–480 × 10^3^. All three targeting ligands were added to lipids at 4% (m/m). If we assume that ligands are incorporated into the niosome in stoichiometric proportions, they can replace 4% of the lipids. Based on this and the molecular weights of the 6-O-palmitoyl-L-ascorbic acid and the dodecanoyl leucine ligands, which are comparable to that of lipids, 10–20 × 10^3^ ligands/NP can be incorporated. Because the molecular weight of PEG-GSH is much higher, we calculated fewer, about 1.4–2.8 × 10^3^ molecules/NP. Finally, we estimated that the ligand ratio in the case of ascorbic acid and leucine was 4–4% and 0.5% for GSH as compared to the total lipid head groups.

### 3.2. Effect of Nanoparticles on the Impedance of Primary Rat Brain Endothelial Cells

**Figure 2:**
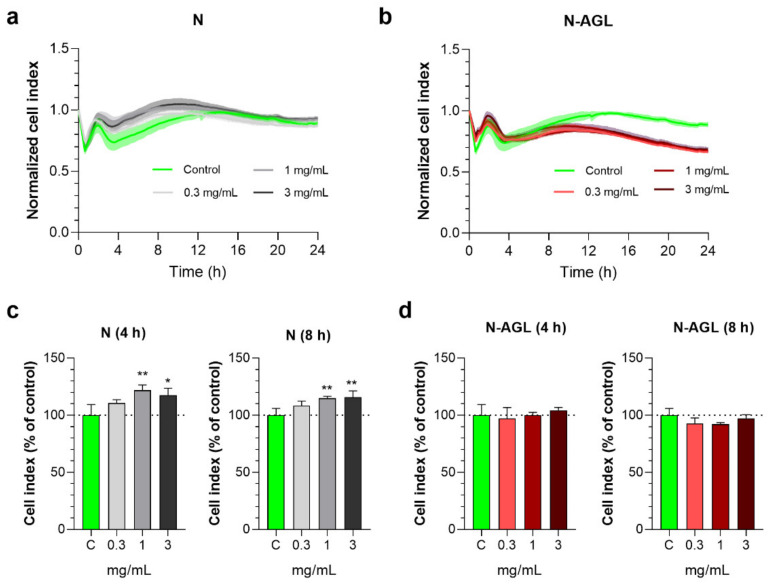
Effect of non-targeted (N) and ascorbic acid-glutathione-leucine-targeted (N-AGL) nanoparticles on the impedance of primary rat brain endothelial cells monitored by real-time measurements. Kinetics of brain endothelial cell responses to (**a**) N and (**b**) N-AGL nanoparticle treatments for 24 h. Values presented are means ± SD and are given as cell index. Impedance of brain endothelial cells treated with N (**c**) or N-AGL (**d**) after 4 or 8 h. Values presented are means ± SD and are given as a percentage of culture medium-treated control. Statistical analysis: one-way ANOVA followed by Dunnett’s post-test; * *p* < 0.05, ** *p* < 0.01, compared to the control group; *n* = 6–8.

We monitored the response of brain endothelial cells to incubation with NPs at 0.3, 1 or 3 mg/mL concentrations for 24 h by real-time impedance measurements (Figure 2a,b). At 4 and 8 h, the cell index values measured by impedance in the N and N-AGL groups were not reduced as compared to the control group receiving culture medium (Figure 2c,d), reflecting that cellular viability was not decreased, in concordance with our prior studies [12,13]. As a safe concentration for the non-targeted and targeted NPs, the 1 mg/mL concentration was selected for the subsequent experiments.

### 3.3. Examination of the Cellular Uptake of NP Cargo and the Mechanism of the Uptake Process

**Figure 3:**
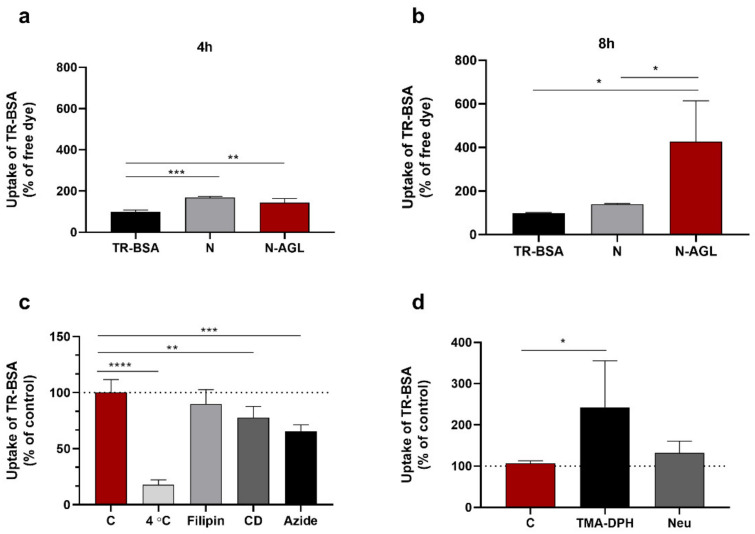
Cellular uptake of Texas red labeled albumin (TR-BSA), and TR-BSA loaded into non-targeted (N) and ascorbic acid-glutathione-leucine-targeted (N-AGL) nanoparticles in cultured primary rat brain endothelial cells after (**a**) 4 h and (**b**) 8 h of incubation. Values presented are means ± SD and are given as a percentage of the TR-BSA group at both time points. Statistical analysis: unpaired *t*-test; * *p* < 0.05, ** *p* < 0.01, *** *p* < 0.001 compared to the TR-BSA groups; *n* = 6. (**c**) Effect of temperature, endocytic inhibitors filipin (5 µg/mL) and cytochalasin D (CD; 0.125 µg/mL) or metabolic inhibitor sodium azide (azide; 1 mg/mL) on the cellular uptake of TR-BSA loaded N-AGL (4 h, 37 °C). Values presented are means ± SD and are given as a percentage of the control group (C; at 37 °C without treatment). Statistical analysis: one-way ANOVA followed by Dunnett’s posttest; ** *p* < 0.01, *** *p* < 0.001, **** *p* < 0.0001 compared to the control group; *n* = 6. (**d**) The effect of neuraminidase (Neu, 1 U/mL) and TMA-DPH (30 mM) on the uptake of TR-BSA loaded N-AGL in rat brain endothelial cells. Values presented are means ± SD and are given as a percentage of the control group. Statistical analysis: one-way ANOVA followed by Dunnett’s posttest; * *p* < 0.05 compared to the control group; *n* = 6.

Significantly increased entry of the cargo TR-BSA encapsulated in N or N-AGL was seen at the 4-h time point compared to the free cargo (Figure 3a). After 8 h, the TR-BSA uptake in the N-AGL group was significantly elevated (N-AGL: 426%): we found more than four times the difference compared to the cargo-only group and three times increase compared to the non-targeted NP group (N: 140%, Figure 3b).

To test the temperature dependence of the cellular uptake of the NPs, cells were treated with N-AGL at both 37 °C and 4 °C (Figure 3c). Decreased cargo uptake (18%) was seen in RBECs at 4 °C as compared to the control group at 37 °C (Figure 3c). In order to investigate if endocytosis is contributing to the cellular uptake of NPs, pharmacological blocking of endocytic pathways by filipin (lipid raft/caveolae-mediated endocytosis) or cytochalasin D (all endocytic pathways by inhibiting actin polymerization) was used. The uptake of the TR-BSA cargo from N-AGLs decreased significantly when brain endothelial cells were pre-treated with these inhibitors (filipin: 89%, cytochalasin D: 78% as compared to the control group), suggesting a role for endocytosis in the uptake of our NPs (Figure 3c). Sodium azide is an inhibitor of ATP synthesis [28]. Cotreatment of the RBECs with metabolic inhibitor sodium azide and N-AGL (4 h, 37 °C) resulted in a significantly lower cellular entry of the cargo (Azide: 66%; Figure 3c).

To reveal the role of the brain endothelial surface charge in the NP uptake process, we modified the surface zeta potential of RBECs. Negatively charged sialic acid residues were removed from the glycocalyx by digestion with neuraminidase (Neu) enzyme, or the cell layers were treated with the cationic lipid, TMA-DPH [12,29]. Surface charge alterations in the cells significantly increased the uptake of NPs (Figure 3d). We found 142% and 32% elevations after treatment of RBECs with TMA-DPH or neuraminidase, respectively, as compared to the control group.

**Figure 4:**
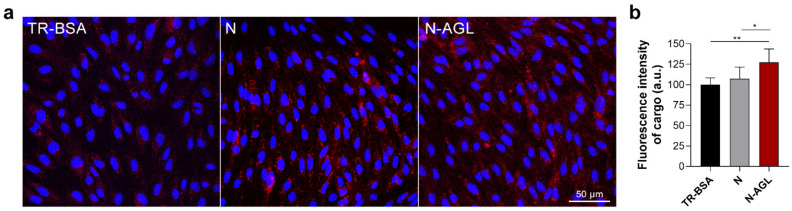
(**a**) Confocal microscopy images of cultured RBECs incubated with Texas red-labeled albumin (TR-BSA; red fluorescence), non-targeted (N) or ascorbic acid-glutathione-leucine-targeted (N-AGL) nanoparticles. Cell nuclei are shown in blue. Scale bar: 50 μm. (**b**) Fluorescence intensity evaluation of TR-BSA, N or N-AGL treated cells. (24 h, 37 °C). Values presented are means ± SD and are given as arbitrary units (a.u.), shown as percentage of the TR-BSA group. Statistical analysis: one-way ANOVA followed by Dunnett’s posttest; * *p* < 0.05, ** *p* < 0.01, compared to the TR-BSA group; *n* = 6.

The cellular uptake of the TR-BSA cargo, giving a red fluorescent signal, was visualized in RBECs by confocal microscopy (Figure 4a). Intensive red fluorescence was detected in cells treated with triple-targeted N-AGL niosomes indicating uptake of the cargo (24 h, 37 °C). The entry of the TR-BSA cargo to brain endothelial cells was much less in the non-targeted NP group and even less when TR-BSA was given to the cells without encapsulation (Figure 4a). In concordance with the results of our uptake experiments, the image analysis revealed that fluorescence intensity of RBECs incubated with triple-targeted N-AGL nanoparticles significantly increased compared to the TR-BSA or N groups (Figure 4b).

### 3.4. Measurement of Plasma Membrane Fluidity after Treatment with Nanoparticles

**Figure 5:**
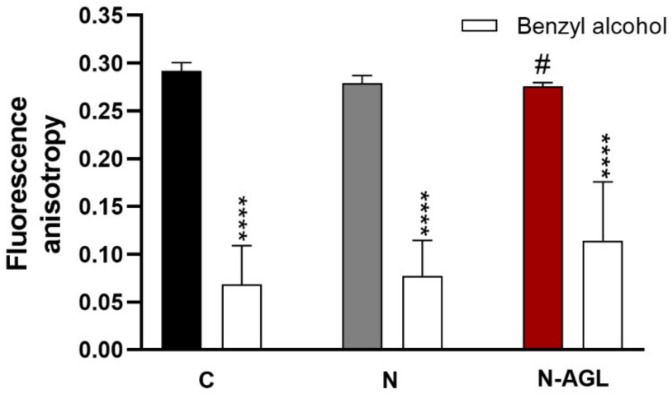
Plasma membrane fluidity measured by fluorescence anisotropy in living brain endothelial cells treated with non-targeted (N) or ascorbic acid-glutathione-leucine-targeted (N-AGL) nanoparticles and membrane fluidizer benzyl alcohol (30 mM). Values presented are means ± SD. Statistical analysis: two-way ANOVA, Bonferroni posttest. ^#^ *p* < 0.05, all groups were compared to medium treated control (C); **** *p* < 0.0001, compared to first column of each group, *n* = 3.

The plasma membrane fluidity of RBECs, determined by fluorescence anisotropy according to our previously published method [12,23], was significantly decreased after 4 h treatment with N-AGL niosomes (Figure 5), indicating increased cell membrane fluidity due to a possible fusion process between the cell membrane and the nanovesicles. As a reference molecule to cause maximal membrane fluidity, benzyl alcohol (30 mM, 3 min) was used, which decreased the fluorescence anisotropy values in all groups (Figure 5).

### 3.5. Permeability of the NP Cargo across the Co-Culture Model of the BBB

**Figure 6:**
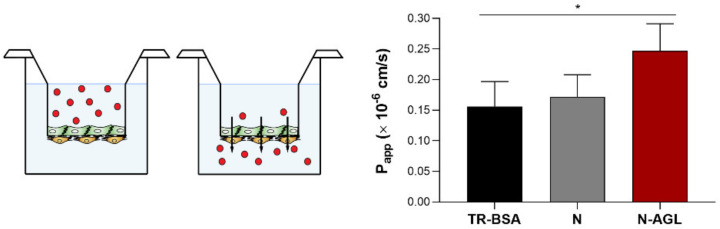
Permeability of the cargo Texas red-BSA (TR-BSA) alone and encapsulated into non-targeted (N) and ascorbic acid-glutathione-leucine-targeted (N-AGL) nanoparticles across the BBB co-culture model. Papp: apparent permeability coefficient. Values presented are means ± SD. Statistical analysis: one-way ANOVA followed by Dunnett’s posttest; * *p* < 0.05 compared to the TR-BSA group; *n* = 4.

The P_app_ of the TR-BSA cargo across the BBB model was 0.15 × 10^−6^ cm/s (Figure 6). The encapsulation of the cargo in non-targeted nanoparticles did not change its permeability through the brain endothelial cell layers (N: 0.17 × 10^−6^ cm/s). Labeling the NPs with three different types of ligands simultaneously resulted in a significant increase in the penetration of cargo across the BBB model (N-AGL: 0.24 × 10^−6^ cm/s). In the case of the N-AGL group, the amount of TR-BSA cargo that crossed brain endothelial cells was elevated 1.6-fold compared to the cargo group (Figure 6). The large hydrophilic FD70 is known to only cross the BBB paracellularly; therefore, its penetration reflects the opening of the paracellular route sealed by TJs. We observed no statistically significant difference in the permeability of FD70 when co-administered with TR-BSA (94 ± 31%), N (111 ± 18%) or N-AGL (118 ± 25%) groups, compared to the control group only receiving FD70. From these data, we conclude that there were no interactions between NPs and TJs that compromised TJ integrity during the permeability experiment.

### 3.6. Visualization of the NP Cargo in Midbrain Organoids

**Figure 7:**
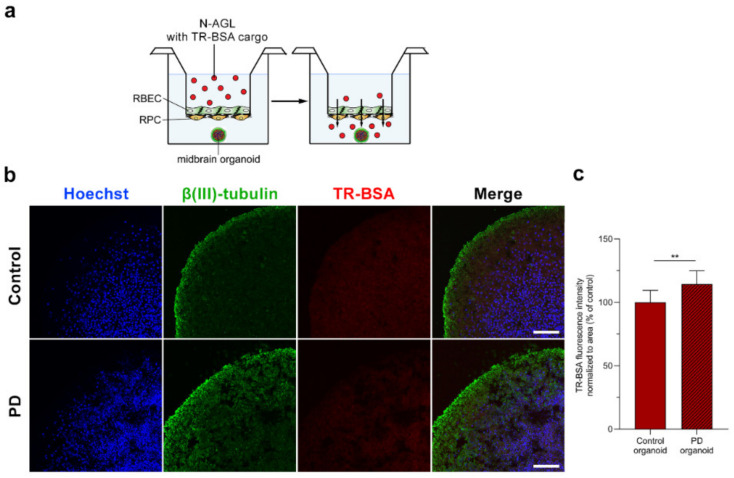
Entry of ascorbic acid-glutathione-leucine-targeted, Texas Red-BSA (TR-BSA; red) loaded nanoparticles (N-AGL) into midbrain organoids after passage across the BBB model. (**a**) Schematic drawing of the experiment. (**b**) Representative confocal microscopy images of midbrain-specific organoids derived from Parkinson’s disease patients (PD) and healthy control (Control). Neurons were immunostained with βIII-tubulin (green), and cell nuclei were stained with bis-benzimide (blue). Bar: 100 μm. (**c**) Fluorescence intensity evaluation of the red channel of the images of control and PD midbrain-like organoids indicating TR-BSA entry. Values presented are means ± SD and are given as arbitrary units (a.u.). Statistical analysis: Student’s *t*-test; ** *p* < 0.01, *n* = 8–10.

We not only investigated the penetration of the NPs across the BBB model, but after passage across the brain endothelial cell layers, we also visualized the entry of the TR-BSA cargo into midbrain organoids by confocal microscopy (Figure 7a,b). We detected the red fluorescent signal of the cargo of the triple-targeted N-AGL in control and PD organoids (Figure 7b). Analysis of the fluorescence intensity in the red channel of the confocal images showed that the entry of the NP cargo into PD organoids was higher compared to the control organoids (Figure 7c).

## 4. Discussion

Among the age-related neurodegenerative diseases, Parkinson’s disease (PD) affects about 8 million patients globally and has a huge impact on healthcare, society and economy [30]. The prevalence of age-related neurodegenerative disorders is increasing, not only because the elderly population has increased, but also because lifestyle greatly affects vascular function, including the cerebrovasculature [1]. Therapies targeting CNS disorders are hampered because protective mechanisms at the level of BBB prevent the entry of potential new drugs and biopharmaceutics to the CNS [31]. To solve this problem, therapeutic molecules may be encapsulated into nanovesicles that will carry them across the BBB. For efficient drug delivery, several factors need to be taken into account when a nanocarrier system is developed [32]. One of the most important factors is the surface functionalization of NPs with ligands of surface molecules of the BBB. Specific targeting can be achieved through exploiting the physiological transport pathways of the BBB, like the receptor-mediated transport pathway or the carrier-mediated transport systems [1].

### 4.1. Selection of Targeted Ligands

SLCs are a superfamily of carrier-mediated transporters of the BBB, which transport a broad spectrum of ligands, including hexoses, vitamins, amino acids, anions and cations [10]. However, SLCs are still underrepresented as potential targets of therapeutic drugs [33] or as potential targeting ligands for NPs designed for brain delivery [34]. In the present study, we selected three different ligands: ascorbic acid, leucine and glutathione, to target the SLC and nutrient transporters of the brain endothelial cells to enhance the transfer of NPs across the BBB. In concordance with our hypothesis, triple labeling of NPs with ligands of BBB transporters (N-AGL) elevated not only the uptake of the large biomolecule albumin cargo in RBECs but also the penetration across the BBB model. Moreover, we could demonstrate the entry of the NP cargo into midbrain organoids after crossing the BBB model.

Glutathione is widely used as a targeting ligand of NPs to cross the BBB in cell culture, animal and clinical studies [9]. Our group demonstrated that not only glutathione [35], but also the combination of alanine and glutathione increased the uptake and permeability of the NPs across a BBB culture model [12,13] and the entry of a protein cargo into the cells of the neurovascular unit, including glial cells and neurons [13]. In addition, with biophysical methods, we also revealed that PEG-GSH, but not PEG alone, binds to the membrane of living brain endothelial cells with an adhesion force in the low pN range [14]. The present results on the increased uptake and permeability measured in the N-AGL group is in agreement with our previous observations [12,13].

Ascorbic acid is an essential vitamin with antioxidant properties necessary for the development and functional maturation of neurons [36] with a 10-fold higher concentration in the CNS as compared to the periphery. The reduced ascorbic acid and the dehydroascorbic acid are the two biologically active forms of vitamin C. SVCT2 is highly expressed at both the blood-brain and the blood-cerebrospinal fluid barriers, and the latter is responsible for the transport of vitamin C to the CSF and brain [37]. The genes of this transporter, *Svct1* and *2*, are expressed at the BBB in mice [38], and culture conditions induced the upregulation of *Svct2* in cerebral capillary endothelial cells [39]. The oxidized form of vitamin C, dehydroascorbic acid, is transported by GLUT1 into the brain at the level of both major CNS barriers [37]. In our previous study, we confirmed that the *Glut1* gene showed the highest mRNA expression level among the several glucose transporters, which were expressed in our BBB co-culture model [7]. Therefore, GLUT1 transporters act as therapeutic targets and provide routes of entry for drug delivery by targeted NPs to the brain for the treatment of neurological and neurovascular conditions [40]. In addition to glucose, it is also widely reported that ascorbic acid derivatives as ligands of GLUT1 transporters have a superior brain targeting efficiency [40]. We found few studies to examine ascorbic acid as a targeting ligand of NPs for brain delivery. Ascorbic acid decoration of polymeric NPs containing galantamine enhanced the cellular uptake of NPs in SVCT2 transporter expressing mouse fibroblasts [41]. In the same study, the higher therapeutic effect of galantamine loaded into ascorbic acid conjugated NPs was demonstrated in rats by behavioral tests, and in a biodistribution experiment, the highest concentration of the peptide in the brain was measured for the targeted NP group. In another study, liposomes dual-targeted with glucose and vitamin C (Glu-Vc) effectively delivered paclitaxel both into the cells and brain [42]. The cellular uptake of Glu-Vc-liposomes in rat C6 glioma cells overexpressing GLUT1 and SVCT2 was significantly higher than that of the paclitaxel cargo alone. The Glu-Vc modified liposomes showed superior targeting ability to glioma in vivo as compared to paclitaxel [42]. It should be noted that neither of these studies used brain endothelial cells as a BBB model to test vitamin C-targeted NPs. In our present study, using a co-culture model of the BBB, we found a 3-fold increase in the uptake at the 8-h time point and 1.4-fold elevation in the permeability of the TR-BSA cargo encapsulated to N-AGL, as compared to non-targeted NPs.

Leucine is an essential amino acid used in the biosynthesis of proteins, which is transported by LAT1, an amino acid transporting SLC, showing a very high gene expression level in human brain microvessels [43]—about 100-fold higher than in peripheral microvessels. In our previous gene expression study, LAT1 had one of the highest mRNA expressions among the BBB influx transporters [7]. LAT1 participates in the transport of the clinically used medicines L-Dopa, melphalan, gabapentin and baclofen to the brain [10]. In our previous studies [12,13], we identified the amino acid alanine as a potent BBB targeting molecule of NPs. Nanovesicles decorated with alanine as one of the targeting ligands elevated the uptake of the NP cargo not only into cultured brain endothelial cells, but also into brain pericytes, astrocytes and neurons [13], and brain entry of NPs in mice [12]. In the present study, leucine was used as a targeting ligand of NPs for the first time as part of a triple combination also containing ascorbic acid and glutathione. 

### 4.2. Mechanism of the Cellular Uptake of Triple-Targeted NPs

The metabolic inhibitor sodium azide and the low-temperature condition decreased the uptake of TR-BSA cargo in the N-AGL group, supporting our hypothesis that entry of targeted NPs into cells is an energy-dependent active process. This finding is in concordance with our previous experiments with dual-targeted NPs [12,13]. We also demonstrated that the uptake of the targeted NPs involves endocytosis, using well-known inhibitors of this process. Cytochalasin D or filipin pretreatment of brain endothelial cells reduced the cellular entry of the cargo of N-AGLs that indicate that endocytosis contributes to the cellular uptake of targeted NPs, in concordance with our previous studies [12,13]. 

Among the BBB features, the negative surface charge of brain endothelial cells as an electrostatic barrier influences the transport of NPs across the BBB [44]. This highly negative surface charge is composed of the negatively charged cell membrane lipids and the glycocalyx on the surface of endothelial cells composed of proteoglycans and glycosaminoglycans [44]. We modified the surface charge of endothelial cells with neuraminidase, which digests the negatively charged sialic acid residues and the TMA-DPH cationic lipid, which inserts into the plasma membrane [29]. When we turned the surface charge of brain endothelial cells more positive, the cellular uptake of the cargo of triple-targeted NPs was significantly increased as compared with the control non-treated culture group. We measured similar changes in our previous study with dual-targeted NPs [12]. Our results demonstrate that the surface charge of the brain endothelial cells can be modulated by modification of plasma membrane lipid composition or the glycocalyx, and this electrostatically negative surface regulates the uptake of NPs with a negative charge. 

The permeability of drugs across biological barriers is increased by different surfactants [45]. Surfactants can incorporate into the cell membranes and can affect the physical properties or the permeability of plasma membranes or cause cell toxicity by membrane solubilization [46]. One of the main components of our NPs, solulan C24, which belongs to the polysorbate surfactants, elevated the penetration of the hydrophilic metformin across Caco-2 epithelial cell layers [47]. In our previous studies, we revealed that sugar esters, non-ionic surfactants, increased the plasma membrane fluidity of epithelial cells and enhanced the permeability of drugs across the cell layers [23]. We measured a decreased fluorescent anisotropy in brain endothelial cells treated with triple-targeted NPs, indicating that plasma membrane fluidity was elevated due to the fusion of the NPs with the cell membranes. We also found increased cell membrane fluidity in brain endothelial cells treated with dual-targeted NPs in our previous work [12].

In our previous study [12], we compared the effectivity of single-targeted NPs with dual-targeted nanoparticles. The dual-labeling of NPs with ligands of BBB transporters (alanine and glucopyranose and alanine and glutathione) elevated the cargo uptake in brain endothelial cells and penetration across the BBB model as compared to both single ligand-targeted or untargeted NPs. The significantly higher brain uptake of cargo in an animal study in the case of NPs labeled with two different ligands of solute carriers at the BBB (alanine and glucopyranose) indicated that targeting more than one transporter at the BBB can be more effective [12]. Based on our previous and present results, we suggest that labeling the surface of NPs with more than one type of ligand-targeting transporters and carriers at the BBB could increase the specificity of brain targeting and result in stronger docking to the brain endothelial cell surface, promoting NP endocytosis as well as fusion with the plasma membrane.

## 5. Conclusions

In this in vitro study, we demonstrated that labeling nanovesicles with the combination of the tripeptide glutathione, the vitamin ascorbic acid and the amino acid leucine increased the cellular uptake of a large protein cargo by more than 3-fold as compared to non-targeted nanoparticles. Moreover, we revealed that the cellular uptake of NPs is energy-dependent and is partially mediated by endocytosis. Finally, we demonstrated the ability of our triple-targeted NPs to deliver the protein cargo into midbrain organoids after crossing a co-culture model of the blood-brain barrier. Our data suggest that ascorbic acid, leucine and glutathione triple-labeling of NPs can promote the transfer across the BBB and brain delivery of molecules.

## Data Availability

The data presented in this study are available on request from the corresponding authors.
